# Correlation of Oxidative Stress Biomarkers and Hematological Parameters in Blood Cancer Patients from Sardinia, Italy

**Published:** 2019-04-01

**Authors:** Ioannis Tsamesidis, Antonella Pantaleo, Anna Pekou, Amrita Gusani, Stavros Iliadis, Kali Makedou, Alessia Manca, Antonio Carruale, Eugenia Lymperaki, Claudio Fozza

**Affiliations:** 1 Department of Biomedical Sciences, University of Sassari, Sassari, Italy; 2 Department of Biochemistry, Aristotle University of Thessaloniki, Thessaloniki, Greece; 3 Department of Pharmacy, University of Portsmouth, Portsmouth, UK; 4 Department of Medicine, Aristotle University of Thessaloniki, Thessaloniki, Greece; 5 Department of Clinical and Experimental Medicine, University of Sassari, Sassari, Italy; 6 Department of Medical Laboratories, Alexander Technological Educational Institute of Thessaloniki, Thessaloniki, Greece

**Keywords:** Oxidative stress biomarkers, Total antioxidant capacity, Malondialdehyde, Reactive oxygen species, Hematological malignancies, Sardinia

## Abstract

**Background: **Over the last few decades, there has been a dramatic increase in hematological malignancies (HMs) in the population of Sardinia. It is accepted that oxidative stress biomarkers have been demonstrated to be prognostically important in various neoplastic diseases. The aim of this study is to evaluate serum vitamin E, total antioxidant capacity (TAC), Malondialdehyde (MDA) and reactive oxygen species (ROS) levels in 80 Sardinian patients with different HMs [acute myeloid leukemia (AML)(n=20), myelodysplastic syndromes (MDS) (n=20), Hodgkin lymphoma (HL) (n=20) and non-Hodgkin lymphoma (NHL) (n=20)] on the day of their diagnosis.

**Materials and Methods:** Samples from all participants were obtained after an overnight fast (at least 10 hours). This study was approved and conducted in accordance with Good Clinical Practice guidelines and the Declaration of Helsinki. Patients and controls provided written, informed consent before entering the study. All study participants’ medical history and their medication were documented upon enrolling.

**Results:** Lower levels of TAC and Vitamin E were observed in most of the studied groups compared to healthy controls (0.41-0.49 mmol/L vs. 0.56 mmol/L) (19.55-28.55 μmol/L vs. 34.51 μmol/L). Moreover, higher average MDA levels were observed in HL and NHL patients compared to healthy controls (16.6 ng/ml-17.8 ng/ml vs. 7.4 ng/ml). Additionally, the ROS values of all studied groups were found elevated. Serum TAC showed significant negative correlations with MDA values (R= -0.51; P<0.001). Statistical significance was observed in all hematological parameters, producing either positive or negative correlation with at least one OS biomarker.

**Conclusion:** The present data suggest that Sardinian patients with HL and NHL on the day of their diagnosis presented the highest OS in comparison to AML and healthy subjects. Moreover, MDS patients presented high OS status. Likewise, our results also indicated that changes in their hematological indices are eminent of their oxidative and antioxidative status.

## Introduction

 The genetic peculiarities *per se* of the Sardinian population have significantly influenced the occurrence of hematological malignancies over the last decades^        1 ^. Meanwhile, recent studies have suggested an increased rate of hematological malignancies in the island^   2 ^. Moreover, several types of genetic mutations in this Mediterranean population result in either deficient enzymes or other mutation types in response to oxidative stress   3^,^^4^. The term ‘oxidative stress biomarker’ has been defined by the National Institute of Health as “a characteristic that is objectively measured and evaluated as an indicator of pathogenic processes, or pharmacological responses to a therapeutic intervention^   5 ^. Several studies have evaluated oxidative stress (OS) biomarkers^   6 ^ for various disorders such as diabetes^   7 ^, malaria    ^8^^-^^10^, heart^   11 ^ and neurodegenerative diseases   ^12^^,^^13^ , along with hemolytic^14^^,^^15^ and neoplastic disorders. The last of which includes hematological malignancies, a diverse group of blood cancers with various etiology, incidence, prognosis, and survival. Among them, myelodysplastic syndromes (MDS) represent a unique scenario as oxidative stress can increase due to both transfusion-dependent iron overload and dyserythropoiesis itself.^16^ Publications from different groups have presented measurements of different oxidative stress biomarkers for different hematological malignancies such as acute lymphoblastic leukemia (ALL), Hodgkin lymphoma (HL) and Myelodysplastic syndromes^17^^-^^23^. Battisti et al. measured plasmatic thiobarbituric acid-reactive substances (TBARS), serum protein carbonylation, whole blood catalase (CAT) and superoxide dismutase (SOD) activities in ALL patients^22^. They found higher TBARS levels and serum protein carbonylation and reduced levels of antioxidants in ALL patients compared to controls. Fracchiolla et al. measured reactive oxygen metabolites (ROMs) levels and total antioxidant capacity (TAC) in onco-hematological patients, and they also confirmed the oxidative imbalance in these group of patients^24^. Despite this, there is not a comprehensive study comparing oxidative stress biomarkers in different hematological malignancies. It is already known that in most of the hematological malignancies, hematopoietic cells can be exposed to a wide spectrum of alterations^25^. At first, excessive oxidative stress damages biomolecules such as DNA, proteins, and lipids, leading to cellular dysfunction and cell death. Accumulation of such damaging effects on individuals can result in diseases such as hematological malignancies. In addition, cells have multiple mechanisms to protect themselves from stress. These mechanisms include apoptosis, DNA repair, cell cycle regulation, and induction of antioxidant and detoxifying enzymes^25^. On the other hand, reactive oxygen species (ROS) activate a cascade of events such as activation of tyrosine kinases and the mitogen-activated protein kinase system, followed by the activation of transcription factor subset^26^^-^^28^. Antioxidants are induced by oxidative stress to act not simply as scavengers of ROS but also as important regulators of oxidative stress responses^29^. Meanwhile, oxidative stress often causes apoptosis, in which mitochondrial control has been known to play an essential role^30^. The dysregulation of antioxidants and apoptosis is deeply involved in the pathogenesis of hematopoietic disorders^31^. Considering the current literature, this study aims to provide more details on this topic, evaluate and compare the antioxidant and oxidative status of Sardinian patients sub-grouped into four prognostically different groups of hematological malignancies. The groups are divided into four categories according to their diagnosis: i) acute myeloid leukemia (AML), ii) Hodgkin (HL), iii) non-Hodgkin (NHL) lymphomas and iv) myelodysplastic syndromes (MDS). As regards the study groups’ oxidative status, direct measurements of ROS and malondialdehyde (MDA), an indicator of lipid peroxidation, were measured. Vitamin E and total antioxidant capacity (TAC) were used to evaluate the antioxidant status.

## MATERIALS AND METHODS

**Data collection**

Samples from all participants were obtained after an overnight fast (at least 10 hours). This study was approved and conducted in accordance with Good Clinical Practice guidelines and the Declaration of Helsinki. Patients and controls provided written, informed consent before entering the study. All study participants’ medical history and their medication were documented upon enrolling.

Table 1 shows the demographic and clinical characteristics of the study groups. A group of 80 Sardinian patients (35 females and 45 males) were examined after blood samples were taken on the day of diagnosis. The results were compared with a sample of forty healthy age-and sex-matched controls, 17 females and 23 males, with an average age of 60.6 ± 10.2 years**.** Most of our patients were characterized by relevant comorbidities (in details, >90% of them for NHL, MDS and AML and >60% of them for HL), so it was impossible to make a formal comparison between the oxidative status of patients with and without relevant concomitant medical conditions.

The study population consisted of 5 groups (Table 1): (All groups are matched for age and sex):

►40 healthy controls

►20 patients diagnosed with Hodgkin lymphoma (HL)

►20 patients diagnosed with non-Hodgkin lymphoma (NHL), 11 with histologically aggressive (DLBCL (diffuse large B cell lymphoma) and mantle) and 9 with indolent (follicular, MALT (mucosa associated lymphoid tissue) or small lymphocyte lymphoma). 

►20 patients diagnosed with acute myeloid leukemia (AML): 11 with an intermediate cytogenetic risk (normal karyotype), 3 unfavorable (monosomy and deletions in chromosomes 5 and 7), 5 favorable with t (8; 21) or t (15; 17), and one unclassified.

►20 patients diagnosed with myelodysplastic syndrome (MDS): 10 RCMD (refractory cytopenia with multilineage dysplasia, 4 RARS (refractory cytopenia with ringed sideroblasts), 3 RAEB (refractory cytopenia with excess of blasts), 2 RAUD (refractory cytopenia with unilineage dysplasia), 15q- syndrome.

**Table 1:** Patient characteristics

**Number of patients**	80
Median age (years)	65.5 (±13.9)
**Sex**	
Male Female	45 35
**AML**	
**ELN stratification risk**^1^ Favorable Intermediate Adverse Not available	3 8 5 4
**MDS**	
** WHO subtype** RA RARS RAUD RAEB 5 q- syndrome	10 4 2 3 1
**R-IPSS** Very low Low Intermediate High Very high	6 9 3 1 1
**NHL**	
**Histology** MALT FL MCL SLL DLBCL unclassified	1 3 2 4 8 2
**Stage** I II III IV not available	1 6 3 7 3
**B symptoms** Yes No not available	4 13 3
**HL**	
**Stage** I II III IV not available	2 8 2 5 3
** B symptoms** Yes No not available	5 12 3

**Abbreviations** **:** DLBCL: diffuse large B-cell NHL, FL: follicular NHL, IPI: International Prognostic Index, MALT: mucosa associated lymphoid tissue NHL, MCL: mantle cell NHL, MZL: marginal zone NHL, NHL: non-Hodgkin lymphoma, SLL: small lymphocyte NHL.

RA: refractory anemia; RARS: refractory anemia with ringed sideroblasts, RAUD: refractory anemia with unilineage dysplasia; RAEB: refractory anemia with excess blasts; R-IPSS: Revised International Prognostic Scoring System.

1 Diagnosis and management of AML in adults: 2017 ELN recommendations from an international expert

**Oxidative stress biomarkers**

A number of assays have been used to measure the levels of oxidative damage in Sardinian patients with various hematological malignancies. These laboratory tests variously measure excretion rates of damaged biomolecules in the blood. Several markers have been measured because of their accurate and clinically relevant approach to evaluate oxidative damage in many different types of damage. All hematological and biochemical parameters were estimated by spectrophotometric methods.

**Total antioxidant capacity (TAC)**

Antioxidant activity was assayed spectrophotometrically, as previously described^        14 ^ using a TAC Kit (Total Antioxidant Capacity Colorimetric assay kit, produced by Cayman Chemical Co., Ann Arbor, USA) with the TEAC method in the clinical chemistry laboratory of the Alexander Technological Educational Institute, Thessaloniki, Greece. 

**Vitamin E**

To determine vitamin E levels, an already described^32^ technique was used within HPLC (High-performance liquid chromatography) system. The alpha-tocopherol was measured by the method of high-performance liquid chromatography reverse phase (RP-HPLC). 

**Malondialdehyde (MDA)**

Lipid peroxidation status in serum was determined by estimating malondialdehyde (MDA) as thiobarbituric acid reactive substances at 532nm, prior to dialysis with high-performance liquid chromatography(HPLC) ^   32 ^ in Laboratory of Biological Chemistry, Medical School, Aristotle University of Thessaloniki, Greece.

**Reactive oxygen species (ROS)**

Intracellular ROS levels in serum were detected using the cell-permeable ROS-sensitive probe 2',7'-dichlorodihydrofluorescein diacetate (CM-H_2_DCFDA) which causes fluorescence upon oxidation. As already described^34^, the fluorescence probe was added to serum and the volume was made up with PBS. The oxidation of CM-H_2_DCFDA was followed by measuring the fluorescence in 96-well black-walled microplates (Corning�, Sigma Aldrich) using a SAFAS Xenius (Monaco). The fluorescence is expressed in “% emission” as determined with the software ‘’Xenius’’. ROS has been expressed as % of the maximal fluorescence obtained with H_2_O_2_ (3 mM).

**Statistical analysis**

Data were analyzed using the SPSS version 22 *.0 Statistical* package. Descriptive statistics presented as mean ± standard deviation and frequencies as percentages. Kolmogorov–Smirnov analysis verified the normality of the data set. Pearson's chi-square test or chi-square test of association was used to discover if there is a relationship between the categorized data, while Fisher’s exact test was used when expected variables were 2% of the total number of variables. Additionally, independent sample t-test was used to compare between means. In all statistical analysis, level of significance (p-value) was set at α=0.05.

## Results

TAC, Vitamin E, MDA and ROS levels (Table 2) in the four patients groups (HL, NHL, AML and MDS) and the healthy group were evaluated among the Sardinian population.

**Hodgkin (HL) and non-Hodgkin lymphoma (NHL)**

The most significant difference between lymphoma samples and healthy controls was seen in patients with HL, where we found lower Vitamin E and TAC levels (average Vitamin E value 19.55 μmol/L vs 34.51 μmol/L of the controls, *P *<0.001) (average TAC value 0.41 mmol/L vs 0.56 mmol/L of the controls, *P *<0.001: a 1.76- and 1.37-fold decrease, respectively in comparison to the controls) and markedly higher levels of MDA (average value 17.76 ng/ml vs 7.52 ng/ml of controls, *P* <0.001: a 2.36- fold increase). 

NHL samples also showed a similar trend for Vitamin E, TAC levels (average Vitamin E value 20.78 μmol/L vs 34.51 μmol/L of the controls, *P *<0.001) (average TAC value 0.45 mmol/L vs. 0.56 mmol/L of controls, *P* <0.001: a 1.66- and 1.25-fold decrease, respectively) and MDA (average value 16.7 ng/ml vs. 7.52ng/ml of the controls, *P* <0.001: a 2.22-fold increase). In particular, among NHL patients greater differences were demonstrated with normal controls in the indolent rather than aggressive subtype. Respectively, an average TAC value of 0.42 mmol/L vs 0.47 mmol/L (*P* = 0.005), and an average MDA value of 17.2 ng/ml vs 16.4 ng/ml (*P* = 0.003), were observed. When comparing all the study groups, ROS levels in both HL (average value 20% vs 3.7% of controls, *P* <0.0001: a 5.40 fold increase) and NHL (average value 13.4% vs 3.7% of controls, *P* <0.001: a 3.62 fold increase) appeared to be in the higher range (Table 2).

**Table 2:** Serum levels of oxidative stress biomarkers in healthy controls and AML, NHL, HL and MDS patients. Mean TAC (mmol/L), Vitamin E (μmol/L), MDA (ng/ml) and ROS (%) values

**Studied groups (n=120)**	**TAC** **(mmol/L)**	**Vitamin E** **(** **μ** **mol/L)**	**MDA** **(ng/ml)**	**ROS** **(%)**
Healthy Controls (n=40)	0.56 (±0.05)	34.51 (±1.45)	7.52 (±0.9)	3.7 (±1.4)
Acute Myeloid Leukemia (AML) (n=20)	0.49** (±0.06)	25.10* (±2.45)	14.9* (±1.1)	10.6* (±1.7)
Non-Hodgkin lymphoma (NHL) (n=20)	0.45* (±0.04)	20.78* (±1.75)	16.7* (±0.7)	13.4* (±0.9)
Hodgkin lymphoma (HL) (n=20)	0.41* (±0.07)	19.55* (±1.65)	17.8* (±0.8)	20* (±2.5)
Myelodysplastic syndrome (MDS) (n=20)	0.55* (±0.03)	28.55* (±1.45)	13.5* (±1.2)	7.5* (±1.5)

**Abbreviations:** TAC: Total antioxidant capacity, MDA: malondialdehyde, ROS: reactive oxygen species; Significant differences between all studied groups (AML, HL, NHL and MDS groups) and healthy controls are marked with *,**.

* P < 0.001,

** P = 0.003, Data were analyzed using a two-tailed, two-sample Student's t-test.

**Acute Myeloid Leukemia (AML)**

Lower values of Vitamin E and TAC were found in the AML group, compared to the healthy group (average Vitamin E value 25.10 μmol/L vs 34.51 μmol/L of the controls, *P *<0.001) (average TAC value 0.49 mmol/L vs 0.56 mmol/l of the controls, *P* =0.003: a 1.14-fold decrease). Indeed, MDA levels were higher (average value 14.9 ng/ml vs 7.52 ng/ml,* P* <0.001: a 1.98 fold increase), without any significant difference between cytogenetic risk groups. In addition, in AML patients there was a moderate negative correlation between MDA and TAC, with r = -0.45 vs. +0.18 compared to the controls. ROS levels of the AML group were significantly higher compared to healthy controls (average value 10.6% vs 3.7%; *P* <0.001: a 2.86-fold increase) (Table 2).

**Myelodysplastic syndromes (MDS)**

Taking into consideration that MDS represent a unique scenario due to the different factors leading to a possible increase in oxidative stress, we compared them specifically with the healthy subjects. MDS patients showed lower levels of Vitamin E (average Vitamin E value 28.55 μmol/L vs 

34.51 μmol/L of the controls, P <0.001) compared to the healthy group (Table 2). Furthermore, there was a moderate negative correlation between MDA and TAC in these patients (R = -0.35; P <0.001). ROS (average value 7.5%) and MDA levels (average value 13.5 ng/ml) were higher compared to the healthy group, (ROS 2.02-fold increase vs controls; MDA 1.79-fold increase vs controls) (Table 2).

**Correlation between OS biomarkers and hematological parameters in Hodgkin and non-Hodgkin lymphoma group**

In Table 3, we present associations between all the tested OS biomarkers (TAC, Vitamin E, MDA and ROS levels) with the hematological parameters of Hodgkin and non-Hodgin lymphoma patients; the two groups with the higher evaluated oxidative stress values on the day of their diagnosis. A statistically significant positive correlation was observed between their bilirubin levels and i) TAC values and ii) Vitamin E levels (R = 0.65; p < 

0.001, R=0.57; p < 0.001); (R = 0.47; p < 0.001, R=0.69; p < 0.001). On the other hand, neutrophil activity seemed to be negatively correlated with TAC activity (R = -0.44 ; p  < 0.001) ; (R = -0.34 ; p  < 0.001) and Vitamin E levels (R = - 0.38 ; p  < 0.001) ; (R = -0.37 ; p  < 0.001), respectively. Furthermore, in the HL group, bilirubin levels presented a statistically significant negative correlation with both MDA and ROS values (R = - 0.75; p < 0.001, R= - 0.49; p < 0.001, respectively). Finally, hemoglobin levels showed a statistical significant positive correlation only with the MDA values (R = 0.33; p < 0.001) in the HL patient group. None of the other measured hematological indices correlated with the OS biomarkers presented statistically significant correlations.

**Table 3:** Correlation between the tested OS biomarkers and hematological indices in Hodgkin and non-Hodgkin lymphoma patients

**Hodgkin and non-Hodgkin lymphoma patients**					
**Pearson's Correlation**		**TAC** **R; p-value**	**Vitamin E** **R; p-value**	**MDA** **R; p-value**	**ROS** **R; p-value**
HGB	HL	0.14;<0.001	0.24; 0.005	0.33; <0.001	-0.09< 0.001
	NHL	-0.13; <0.001	0.18; 0.007	0.07;<0.001	-0.09; 0.07
WBC	HL	-0.36; 0.008	-0.19; 0.007	-0.02; < 0.001	-0.12; < 0.001
	NHL	0.25; <0.001	-0.09; 0.014	0.01; < 0.001	0.37; < 0.001
Bilirubin	HL	0.65; < 0.001	0.57; < 0.001	-0.75 < 0.001	-0.27; 0.04
	NHL	0.47; < 0.001	0.69; < 0.001	-0.39< 0.001	-0.11; < 0.001
Ferritin	HL	-0.11; 0.03	0.12; 0.15	-0.32< 0.001	-0.26 ; 0.05
	NHL	-0.47; < 0.001	0.08; 0.17	-0.08< 0.001	-0.001 ; 0.14
NEU	HL	-0.44; < 0.001	-0.38; < 0.001	0.14< 0.001	-0.20 ; 0.05
	NHL	-0.34; < 0.001	-0.37; < 0.001	-0.25< 0.001	0.27; < 0.001

*Correlation is significant at p value < 0.05 (2-tailed)

Abbreviations: WBC-White blood cells; HGB-Haemoglobin; NEU-Neutrophils; PLT-platelet count; NEU-neutrophil count; LYM- lymphocyte count;

## Discussion

 It has already been demonstrated how in some neoplasms oxidative stress is characterized by an imbalance between the production of ROS and a biological system’s ability to repair itself^35^^,^^36^. This study has the potential to evaluate the oxidant and the antioxidant status of Sardinian patients with different blood and bone marrow disorders. The HL patient group demonstrated the greatest oxidant status, whereas patients with MDS showed the least. In agreement with our study, Bur et al.^20^ also suggest that significant OS exists in HL and especially in patients with more aggressive forms of the disorder. Moreover, high ROS and MDA production in HL patients has been associated with a significant decrease in antioxidant defence mechanisms^37^. Also, our study showed that the bilirubin system of HL patients seems to be affected by their oxidative status. Regarding MDS patients, Pimkova et al.^23^ did not observe increased MDA levels which contrasts with our findings in this group of patients. But, Goncalves et al.^16^ in agreement with our study observed increased ROS levels and consequently the involvement of oxidative stress to the development of MDS. Among all the analyzed hematological malignancies, we found an increase of oxidative damage, although of varying degrees. Our results indicate that the studied parameters of the OS status in human blood are potentially modulated by concomitant neoplastic disorders, suggesting an overall increase in OS. Importantly, the level of the studied markers, as well as the possible correlation between these parameters and their hematological indices in different patients group might have prognostic relevance for healthy subjects. Moreover, it will be worthy, to evaluate possible correlations between oxidative stress biomarkers and iron levels of MDS patients, which has shown to influence survival in patients with low risk- myelodysplastic syndromes^38^. Nowadays, it is well known that in these disorders oxidative stress is dependent on labile iron plasma. In fact, iron overload in these patients derives from both blood transfusions and dyserythropoiesis whereby both factors can contribute to establishing an oxidative status in their cells. For this reason, more data are needed to better understand the mechanisms of hematological improvement and evaluate the oxidative stress markers in MDS patients under different iron chelation therapies^39^.

Our findings may also suggest a possible use of these parameters for screening and increased monitoring of these neoplastic conditions. Further studies are required to determine the source and species of ROS generated by tumor cells and whether the ROS with therapeutic effects originates from the metabolism of normal cells or rather neoplastic cells. The knowledge that OS induces protein carbonylation by inactivating antioxidant enzymes^40^ could be used to investigate an efficient way to boost the endogenous production of antioxidants and incorporate them into diets, thus helping to neutralize the free radicals produced by cellular metabolisms. 

## CONCLUSION

 We suggest the involvement of oxidative stress in HL, NHL, AML and MDS development and prognosis. Patients with HL and NHL presented the worst oxidative status. Moreover, oxidative stress in MDS patients seems to be subtype-dependent. Regarding the tested biomarkers, TAC and MDA may constitute novel markers with value in diagnosis and/or prognosis of these diseases. More studies need to be done in order to clarify the associations between OS biomarkers and hematological parameters in Hodgkin and non-Hodgkin lymphoma group. In conclusion, in all studied groups a negative correlation (R = -0.51; *P* < 0.001) between TAC and MDA levels was observed, whereby MDA values increased as the TAC levels decreased. In addition, lower Vitamin E levels in all studied groups in comparison with the healthy group confirmed the low values of total antioxidants. Furthermore, ROS levels appeared to be significantly higher in all studied groups compared to the healthy group (*P* < 0.001). The present study contributes to a better understanding of the oxidative basis of neoplastic diseases, as multifactorial and heterogeneous diseases.
